# Focal Laser Ablation of Prostate Cancer: Definition, Needs, and Future

**DOI:** 10.1155/2012/589160

**Published:** 2012-05-16

**Authors:** Pierre Colin, Serge Mordon, Pierre Nevoux, Mohammed Feras Marqa, Adil Ouzzane, Philippe Puech, Gregory Bozzini, Bertrand Leroux, Arnauld Villers, Nacim Betrouni

**Affiliations:** ^1^Inserm U703, Université Lille Nord de France, CHRU Lille, 59037 Lille, France; ^2^Department of Urology, CHRU Lille, 59037 Lille, France

## Abstract

Current challenges and innovations in prostate cancer management concern the development of focal therapies that allow the treatment of only the cancer areas sparing the rest of the gland to minimize the potential morbidity. Among these techniques, focal laser ablation (FLA) appears as a potential candidate to reach the goal of focusing energy delivery on the identified targets. The aim of this study is to perform an up-to-date review of this new therapeutic modality. Relevant literature was identified using MEDLINE database with no language restrictions (entries: focal therapy, laser interstitial thermotherapy, prostate cancer, FLA) and by cross-referencing from previously identified studies. Precision, real-time monitoring, MRI compatibility, and low cost of integrated system are principal advantages of FLA. Feasibility and safety of this technique have been reported in phase I assays. FLA might eventually prove to be a *middle ground* between active surveillance and radical treatment. In conclusion, FLA may have found a role in the management of prostate cancer. However, further trials are required to demonstrate the oncologic effectiveness in the long term.

## 1. Introduction

Prostate cancer is the most frequent cancer among men over 50 years old in industrialized countries. With PSA screening, development of new prostate biopsies protocols, and MRI, the accuracy of detection and localization has increased. Also, today a growing number of small-volume and low-grade cancer foci are diagnosed in young healthy men. Standard treatments for prostate cancer such as surgery or radiation involve the whole gland, even if the tumor is localized. Despite their oncologic efficiency, radical treatment modalities are associated with significant morbidity (urinary and sexual dysfunction) and may be linked to useless overtreatment, which affects quality of life of patients diagnosed with very low development of potential small localized tumors.

Today, 94% of low-risk cancers are treated with radical treatment. In 2009, the ERSPC study has proven the value of screening [1]. A gain of survival of 27% among screened men aged 55 to 69 was demonstrated, with an average follow-up of 9 years after diagnosis. But this gain of survival was associated with a high rate of overdiagnosis and overtreatment.

Active surveillance can be an alternative to radical treatment in this indication. However, this strategy induces important psychological stress for patients, and it is often difficult for clinicians to propose this management option to young men with long life expectancies.

Today, a new treatment concept is emerging and is termed *focal therapy*. The challenge of current focal therapy techniques is to treat only localized tumors sparing the rest of the prostate to minimize the potential morbidity.

To be effective, focal therapy must be (1) guided by imaging (to define the exact location of cancer area), (2) able to target only the desired area (dosimetric planning), and (3) followed by surveillance of the untreated areas.

Different energy sources are being developed for this indication, and some are currently under clinical protocols such as cryotherapy, high-intensity focus ultrasound (HIFU), or vascular photodynamic therapy (VTP).

**Figure 1:**
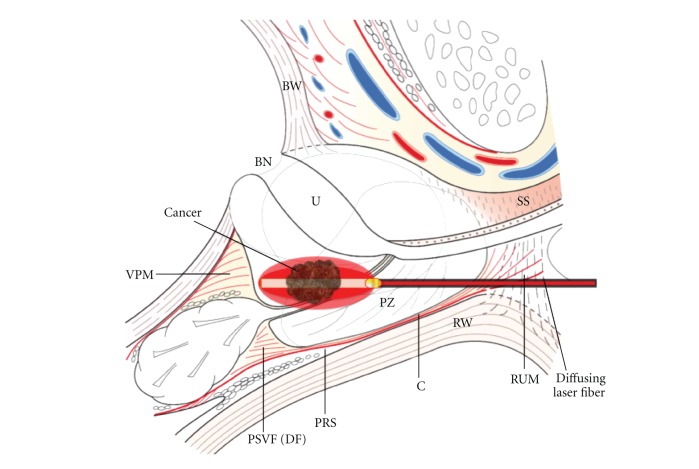
Principle of FLA. The diffusing laser fiber was introduced by transperineal way (schematic midline sagittal section of prostate, bladder, urethra, and striated sphincter). BW: bladder wall; BN: bladder neck; U: urethra; SS: striated sphincter; RUM: rectourethral muscle; RW: rectal wall; PZ: peripheric zone; C: capsule of prostate; DF: Denonvilliers' fascia; VPM: vesicoprostatic muscle; PSVF: posterior seminal vesicle fascia; PPF: posterior prostatic fascia.

Focal laser ablation (FLA) by interstitial thermotherapy could be another modality for prostate cancer focal therapy. This technique presents the following benefits: an ease of use, low cost, and less cumbersome workstation. This paper describes the mechanisms, history, and components of FLA with an account of current clinical experience for prostate cancer. The principle of transperineal FLA is illustrated in Figure 1. 

Relevant literature was identified using MEDLINE database with no language restrictions (entries: focal therapy, laser interstitial thermotherapy, prostate cancer, focal laser ablation) and then by cross-referencing from previously identified studies. Articles were selected by their relevance to the topic. Current clinical trials concerning FLA were found using the database clinical trials conducted in the USA and around the world (http://www.clinicaltrial.gov/).

## 2. FLA Mechanisms and Components

### 2.1. FLA Mechanisms

FLA is defined as the thermal destruction of tissue by laser. For prostate cancer, this denomination is preferred to the other names such as photothermal therapy, laser interstitial tumor therapy, and laser interstitial photocoagulation because it describes both the intention and the treatment [8].

FLA action is based on a photothermal effect. The thermal action results from the absorption of radiant energy by tissue receptive chromophores inducing heat energy in a very short time (few seconds) [18]. This increased temperature may cause irreversible damages and remotely *in vivo* destruction. The thermal effect depends on the amount of heat energy delivered but also on the depth of light distribution. Consequently, the deep tissue damage is dependent on the wavelength of the laser in action. Due to weak absorption by water or hemoglobin, wavelengths between 590 and 1064 nm are classically used to obtain a deeper tissue penetration. 

The extension of thermal tissue damage depends on both temperature and heating duration. Cell viability is in relation with thermostability of several critical proteins. Irreversible protein denaturation may occur around 60°C [19]. While over 60°C, coagulation is quasi-instantaneous, between 42 and 60°C, a thermal damage is obtained with longer heating periods. The area submitted to supraphysiological hyperthermia less than 60°C will develop coagulative necrosis in 24 to 72 h after treatment [6, 20]. Macroscopic appearance of coagulation areas of FLA corresponds to well-demarcated foci of necrosis surrounded by a small rim of hemorrhage with no viable glandular tissue (benign or malignant) after vital staining, based on immunoreactivity with cytokeratin [9, 21].

### 2.2. FLA Material

#### 2.2.1. Computed Dosimetric Planning

Pretreatment dosimetric planning of FLA requires three steps to predict the extent of the coagulated necrosis [18]. An optothermal model of FLA consists in calculating light distribution, temperature rise, and the extent of thermal damage. Light distribution could be obtained using the Monte Carlo simulation to estimate photons distribution in irradiated tissue. This process is based on tissue optical properties at the used laser wavelength.

Absorption of light in tissue causes a local elevation in temperature. Tissue heat transfer due to the energy of light deposited is described by the bioheat transfer equation (Pennes' equation).

Thermal damage in cells and tissue can be described mathematically by a first-order thermochemical rate equation, in which temperature history determines damage. Damage is considered to be a unimolecular process, where native molecules are transformed into a denatured/coagulated state through an activated state leading to cell death. It is calculated from the Arrhenius law [22].

Previous theoretical models of prostate treatment have generally assumed threshold damage temperatures of 50°C. These values are based on studies involving exposure durations of about seconds or greater. For instance, histological evaluation performed by Peters et al. showed that the thermal-injury boundary can be predicted from a threshold-maximum temperature of approximately 51 degrees °C [3, 20, 23]. 

#### 2.2.2. Laser Sources

As it was reported above, wavelengths in the range of 590 to 1064 nm are the most adequate to induce a maximal photothermal effect in human tissue. At the beginning of interstitial laser coagulation development, Nd:YAG laser (1064 nm) was used. This laser source allowed deep penetration into the tissue (10 mm); however, this kind of material is cumbersome due to the need for cooling systems.

In 1998, with the development of laser for benign prostate hypertrophy (BPH) treatment, small diode lasers appeared allowing interstitial laser coagulation at 830 nm with transurethral application of diffusing fibers [24, 25]. Thereafter, these diode lasers were used for hepatic and brain tumors treatment in near-infrared (800–980 nm) [26]. With these wavelengths, while tissue penetration is weaker (5 mm) in comparison with Nd:YAG laser, these diodes present an excellent energy efficiency permitting the minimization of their cooling system. Improvements in the design of high-power diode laser sources have made medical laser systems smaller, more portable, more powerful, and less expensive than previous generations. Since 2011, diode laser emitting at 1064 nm ± 10 nm has been proposed and could replace the Nd:YAG laser.

#### 2.2.3. Optical Fibers

Light is delivered via flexible quartz fibers of diameter from 300 to 600 *μ*m. Conventional bare-tip fibers provide a spherical lesion of about 15 mm diameter at their ends, but have been largely replaced by interstitial fibers consisting of cylindrical diffusing tips of 10 to 40 mm of length. These needles provide larger ablative area of up to 50 mm [27, 28].

#### 2.2.4. Temperature Monitoring

**Two Different Temperature Monitoring Set-Ups Are Proposed**

(i) Thermocouples placed at the laser probe are used to control the laser power in the adaptive monitoring mode. Usually, the initial laser power is set at 15 W for each fiber and the control temperature is set at 100°C. As the temperature measured by the laser fiber probe quickly increases to 100°C, the power delivered by the laser quickly decreases and stabilizes at about 2 W [11].

(ii) Fluoroptic temperature probes used to control the temperature of specific structures. Due to their technology theses probes (Model 3100, Luxtron Corp., Santa Clara, California) are insensitive to the magnetic fields. These probes can be used to validate the measurements performed by MR thermometry. Usually, they are placed at the expected ablation boundary to ensure that therapeutic temperatures (55°C or greater) are reached at the target borders and to control that near-critical structures remain unaffected by heat (by maintaining temperatures less than 42°C) [29].

#### 2.2.5. Real-Time MRI Control

Multiparametric MRI devices are valuable tools for laser fiber guidance and control of coagulative necrosis after FLA [3, 11]. As previously demonstrated for other organs, the extent of tissue necrosis is visible in MRI (T1 weighted spin echo and FLASH sequences) [29, 30]. During the procedure, real-time 3D temperature maps could be obtained using the proton resonance frequency (PRF) shift. For MRI thermometry, a gradient-recalled echo pulse sequence is rapidly repeated during FLA procedure. With dedicated software, the acquired MR images are analyzed in real time to estimate the thermal changes; computation of the ablation zone maps was done using the Arrhenius model of thermal tissue ablation. 

## 3. Experimental Trials

### 3.1. Primary Development

The use of lasers to coagulate tumors was first proposed in 1983 by Bown [31]. Application for prostate ablation started in 1993-94 [2, 12].

With development of laser treatment for BPH, feasibility of FLA was established in canine model with a Nd:YAG laser (1064 nm) [2]. Johnson et al. reported an immediate well-demarcated area of acute coagulative necrosis surrounding each laser fiber pathway. A continuous and progressive enzymatic tissue liquefaction led within days to the development of central necrotic cavities.

Amin et al. reported the first clinical application of FLA for local recurrence of prostate cancer after external radiotherapy [12]. The procedure was performed under intravenous sedation with 805 nm diode laser. Laser fibers were inserted transperineally using 18G needles under US-guidance and CT-scan control. A bladder and urethral cooling was performed using continuous saline solution perfusion with triple-lumen urinary catheter. Procedure was well tolerated by patient with hospital discharge 24 h after treatment. A nonenhancing zone corresponding at ILC area was visible in CT-scanning control at 10 days. Control biopsies at 3 months confirmed presence of coagulative necrosis in treated area and revealed cancer cells in other untreated area. A second laser treatment was performed without major side effect [2, 12]. At that time, development was limited by accuracy to localize the cancer areas on pre-operative evaluation, computer dosimetric planning, and imagery follow-up.

### 3.2. Preclinical Developments

Table 1 summarizes the different preclinical publications dealing with FLA.

In 2000, Peters et al. described MRI-guidance and real-time thermometry for FLA in an *in vivo* canine prostate model [3]. They used an 830 nm diode laser (Indigo, Ethicon EndoSurg) with a quartz-clad diffuser at the end of laser fiber. Developments in MR imaging allowed accurate fiber positioning and obtaining quantitative 3D maps of *in vivo* temperature during the photothermal treatment. Animals were sacrificed at 4 and 24 h after the procedure. On histologic sectioning, the necrotic area was surrounded by hemorrhage and acute inflammation. In light microscopy, intact viable cells were described in the necrotic area of animal sacrificed after 4 h. 

As previously described, these tissue areas exposed to supraphysiological hyperthermia (between 42 and 60°C) take from 24 to 72 hours for the full extent of lethal thermal damage to be revealed by necrosis. The cells appeared intact at this 4 hr time point because their intrinsic lytic enzymes have been thermally denatured and will likely persist until new blood vessels bring inflammatory cells to invade and digest the necrotic tissue. Consequently, on posttreatment dynamic MRI control, the visible margins of thermal necrosis were smaller than the histological finding. The authors concluded that thermal damage planning using MRI thermometry was needed.

In another study, after *ex vivo* calibration, Fuentes et al. performed FLA at 980 nm under MRI in canine prostate model (2 animals) [4]. They realized 1.5 Tesla MRI real-time thermometry, heat shock protein (HSP) expression, and cellular damage planning. The laser procedure consists in heating at 5 Watts for 90 seconds.

The objective of obtaining a visible necrosis over 12 mm of diameter was reached on immediate histopathologic examination. The authors concluded there was good correlation between planning and histopathologic observations. These findings were confirmed by Stafford et al. working on a canine model (5 dogs with healthy prostate and 2 immunosuppressed dogs with prostate cancer) [5]. Different levels of energy were used at 980 nm. This study demonstrated the feasibility of MRI laser fiber guidance with template. The laser fiber was placed in target site with accuracy (mean standard deviation = 1.1 mm ± 0.7). The correlation ratio between planning and histopathologic findings was about 0.94.

More recently our research group prove reproducibility of FLA in rat model with heterotopic tumor for one energy level [6]. The volume of visible necrosis in MRI was significantly different between 1 hour and 48 hours after FLA procedure (*P* < 0.001). This difference was explained by the existence of a noncoagulated degenerative zone surrounding the coagulative necrosis zone in the acute phase, which developed coagulative necrosis after 48 hours as previously described. Histological analysis showed a correlation with the mean necrosis volume obtained by MRI at 48 h (*r* = 0.87). Histopathologic findings were in accordance with cellular damage planning.

In order to get a realistic model with materials presenting optical properties values closer to those of human prostate, Lindner et al. described a prostate phantom gel [10]. This allows the implementation of the FLA under ultrasound, CT, or MRI. The MR thermometry was validated with this model by temperature measures correlation obtained by fluoroptic thermometry (Luxtron Model 3100 optical probes were placed *in situ* in the phantom and could determine the temperature in real time).

In another study, Woodrum et al. have tested FLA at 980 nm on cadaveric model with MRI thermometry and damage planning (Visualase system) [11]. They concluded that MRI real-time thermometry and transperineal fiber guidance thru a template is technically feasible.

## 4. Clinical Trials

Table 2 summarizes the different clinical publications on FLA.

Several clinical studies in North America have recently been reported [9, 13–15]. An initial phase I study (NCT00448695, http://www.clinicaltrial.gov/) was published by the team of Professors Trachtenberg and Haider in Toronto who used a laser diode of 830 nm already used for the treatment of prostatic hyperplasia (Indigo Laser) [13]. After planning on MRI, the laser fibers were placed by a transperineal way; the monitoring of real-time processing was achieved by contrast-enhanced ultrasonography (CEUS). Necrotic lesions were visible on this examination (hypovascular zone), and the volumes obtained were consistent with those obtained on the control MRI.

Postoperative morbidity was negligible. Adverse events reported most frequently consisted of a perineal discomfort (25%) and mild hematuria (16%). Seventy-five percent of patients treated could leave hospital the day after the procedure. At 6 months, there was no significant decrease in erectile dysfunction score (IIEF-5) or worsening of urinary symptoms assessed by the International Prostate Symptom Score (IPSS).

On six-month biopsies, the authors reported short-term oncological results with 67% of patients without recurrence of tumor in the treated area [14]. These results described in phase I trial need to be pondered: the goal of this study was to assess the technical feasibility of FLA procedure. Also, short-term oncologic results could be considered as interesting but still limited by technical problems: in case of residual cancer cells in the ablated zone (33% of patients), the failure of treatment was explained either by poor overlap between pretreatment planification and posttreatment MRI results or by extraprostatic tumour extension to which MRI did not allude on pretreatment or posttreatment scan.

Thus, these limitations came from the poor visualization of the tumor and the difficulty of fiber guidance to the target previously defined. These same limitations also apply for other interstitial techniques (cryotherapy or photodynamic therapy). Advances in MR imaging and those of fiber guidance (nonrigid registration between MRI and ultrasound, real time guidance under MRI, robotic) should resolve these technical gaps.

Two other studies demonstrate the feasibility of focal treatment of cancer by MRI-guided FLA system at 980 nm with Visualase system (NCT00805883 and NCT01094665) [11, 28]. In the phase I trial NCT00805883 including 4 patients, the laser fiber guidance was performed as previously reported [9]. A radical prostatectomy was performed one week after the FLA procedure. Analysis of surgical specimens concluded for good correlation between the volumes of thermal damage visible on MRI and those actually recorded on the vital stain histopathological parts set (Pearson's coefficient *R* = 0.89). In the thermal ablation zone, lack of viable tumor cells seen after immunostaining for cytokeratin 8 validates the scientific relevance of this minimally invasive treatment modality [9, 21].

In phase I trial NCT01094665, Raz et al. described fiber guidance under 3D MRI reconstruction by transperineal approach in 2 patients. MRI thermometry and thermal damage planning were calculated using Visualase system. Transrectal CEUS was realized immediately after the FLA procedure, and in case of residual vascularized target tissue, another procedure with new fibers position was performed. The patients were discharged home within 3 h, and no adverse event or complication was noted at ≤1 month following treatment [15]. The same research team described feasibility of robotic MRI-guided FLA in one case [16].

Recently, Woodrum et al. reported the case of one patient with locally recurrent prostate cancer after radical prostatectomy treated by FLA under 3T MRI guidance [17]. Authors reported no change in continence or potency after the MRI-guided FLA procedure.

Today, three phase 1 trials are recruiting in Canada (NCT01094665) and the USA (NCT01192438 and NCT01377753), and many American centers are already equipped with the Visualase system and have started to publish on the FLA technique for focal treatment. In Europe, a pilot study will open soon.

## 5. Discussion

Focal therapy for prostate cancer is recent and controversial in the urological community.

Showing a high rate of overdiagnosis and overtreatment concerning men with low-risk cancers, the results of international studies led to increased interest in alternative strategies and treatment options [1]. Also in selected patients, focal therapy could be an interesting alternative between radical treatment and active surveillance [32, 33]. Although the idea of focal treatment is simple (ablating a specific and previously defined area sparing uninvolved tissue), the application for prostate cancer met some difficulties: criteria of patient's selection for focal therapy, precise localization, visualization and characterization of significant cancer foci, accuracy guidance of ablative energy in the area to be treated, oncologic efficacy evaluation, and finally surveillance modalities.

Nowadays, we are witnessing the concomitant development of the means of detection and the treatment modalities of these small significant cancers. Also, different energy sources are being tested for this indication. If limited short-term oncologic results of prior focal trials concerning cryotherapy or HIFU are encouraging, other ablative modalities such as VTP or FLA have only demonstrated technical feasibility to date [32].

FLA is an underdevelopment minimally invasive technique for *in situ* destruction of solid-organ tumors. Based on the use of low-power laser, which delivers luminous energy using an adapted optical system, FLA produces a coagulative necrosis zone with a controlled volume, reducing the risk of healthy adjacent structures damage (nerves, blood vessels, sphincter) [6, 9, 13–15].

Before the generalization of this concept, many issues have to be addressed. First, accurate localization of the tumor is required. MRI technology is emerging as the most important imaging tool for identifying low-volume prostate cancers, characterizing tumours, helping in patient-risk stratification, and allowing targeted use of biopsy [33, 34]. Accuracy of MRI as just imaging modality was reported in our previous work with radical prostatectomy histopathology correlation in which MRI sensitivity and specificity for identification of significant cancer foci (>0.5 cc) were 86 and 94%, respectively [35]. Other teams also reported similar results. Besides identification, information concerning location and contours of lesions is important to consider an ablative technique. Important knowledge on modelling of cancer morphology such as zone of origin and intraprostatic patterns of spread of organ-confined prostate cancers at histopathology was made available for imaging interpretation and treatment planning decision [36, 37]. In a study by Dickinson et al., when the location is depicted by biopsy and the tumour is visualized by MRI, it accurately denotes the specific location 83% of the time in the peripheral zone for tumors larger than 4 mm in diameter [38]. It should be nevertheless noted that MRI application requires a degree of discipline in its conduct, reporting, and evaluation to obtain homogenous results among centers as emphasized by a recent European consensus meeting [39]. If MR imaging still lacks a strong validation, nowadays it remains the most important available imaging tool for identifying early prostate cancers and enabling focused use of energy ablative modalities.

The second issue concerns the ability to place precisely the laser-diffusing fiber within the target area. Using a brachytherapy-stabilizing apparatus with modified template grid and VariSeed system, Atri et al. were able to target in transrectal ultrasonography a suspicious area visible in MRI after rigid body registration [13]. Also, to compensate prostate deformation using transrectal sonography, the authors planned a target volume four times higher than the MRI suspicious area. In Phase I NCT00448695, Lindner et al. described the same transperineal technique using 3D US and deformable registration for MR images fusion [14]. The authors concluded that improved deformable registration techniques might be able to minimize registration error and improve targeting. The development of such commercial devices with integrated laser is now achieved (e.g., Echolaser by Esaote), but no data are available concerning FLA of prostate application to the best of our knowledge.

 Then, to enhance accuracy and facilitate real-time assessment of lesion size, the same team performed fiber placement manually under MRI procedure [15]. They used an MRI-compatible template grid and multiplanar images to obtain virtual 3D representation of the template with insertion paths and fiber placement within the prostate. Accuracy of MRI-template-based manual targeting was tested in a preclinical study concluding to standard deviation of 1.1 ± 0.7 mm in fiber placement [5]. Now emerges the possibility of robotic guidance under MRI. In recent years, robotic MR-guided biopsy of the prostate has been reported to be of technical safety and a high degree of accuracy in the biopsy needle placement [39, 40]. Lindner et al. described first case of robotic MRI-guided FLA [16]. Moreover the accuracy to place the diffusing part of the laser fiber within the prostate cancer can be improved: the authors demonstrated that the robot can be used to produce oblique insertion angles to provide adequate dose coverage of low-volume tumors or with difficult location (anterior). This placement technology could be used for FLA under 3D-CEUS too but has not been described with this energy source to the best of our knowledge [41].

The third issue is the treatment planning required to optimize therapy parameters to ensure the optimal coverage of the area while sparing surrounding tissue. This issue is challenging and still needs the development of dedicated dosimetric tools as it was the case for radiotherapy and brachytherapy. Recent key advances in MRI allowed us to open some technological locks in FLA monitoring and guidance. With computer modeling development for thermal damage and multiplanar MR temperature imaging, it is now possible to accurately determine the expected thermal necrosis in region of interest and to control in real time the photothermal effect on homogeneous tissue [5, 7, 42]. Commercialisation of integrated system (laser source and fiber, computerized planning, and monitoring solution) has made MRI-guided FLA clinically relevant [14, 15]. However some limits are still present. Principal variability observed between predicted and obtained ablation areas is due to tissue heterogeneity. This heterogeneity is in relation firstly with own relaxation properties of the tissue (dependant on zonal anatomy, presence of tumor, vascularization, etc.) and secondly with changing of the tissular thermal conductivity during temperature increasing. New nonlinear calibrated computational model of the bioheat transfer may provide a reasonable approximation of the laser-tissue interaction, which could be useful for treatment planning in heterogeneous areas such as prostate cancer [4, 7]. Another limitation for thermal necrosis prediction is related with cooled applicators. To avoid charring or photovaporization, most teams are using cooled applicators to maintain a temperature between 60 and 100°C during the heating phase. The use of these types of applicators necessitates computer modeling of the rise in temperature of the *in situ *fluid to reduce systematic errors [4, 43].

In order to reduce morbidity for healthy adjacent structures and reinforce specificity of FLA for cancer cells, recent preclinical development have been described using nanoparticles [44–46]. The goal of this technique is to produce photothermal coagulation in prostate tissue containing nanoparticles by near-infrared (NIR) activation. Although this is not shown specifically for prostate adenocarcinoma cells, the preferential accumulation of nanoparticles in cancer cells compared to healthy tissue has been suggested (passive diffusion in tumour neovasculature by enhanced permeability and retention effect). Also, if this theoretically selective accumulation was confirmed, the NIR illumination could activate a specific coagulation of the cancer cells. Thus, photoactivation of nanoparticles may occur at power levels that do not generate significant damage to healthy tissue. However the absence of toxicity of these nanoparticles must first be proven before considering an application in clinic.

In conclusion FLA is a potential tool for focal therapy of low-risk prostate cancer. Precision, real-time monitoring, MRI compatibility, and low cost of integrated system are principal advantages of this minimal invasive therapy. Feasibilty and safety of this technique have been reported in phase I assays. Further trials are required to demonstrate the oncologic effectiveness in the long term.

## Figures and Tables

**Table 1:** Preclinical publications concerning focal laser ablation.

Reference Year	Type of preclinical model	Wavelength Type of laser source	Energy (Joules) Power (Watt) Time (seconds)	Type of imagery control	Delay between procedure and histopathologic examination	Dimension of thermal necrosis	Conclusions
Johnson et al. 1994 [2]	Dog (x9)	1064 nm (Neodymium YAG)	3000 J 10 W 300 sec	No imagery control	3 hours to 35 days	13–20 × 17–25 mm (median: 15 × 23 mm)	Immediate coagulation > 60°C Progressive coagulation 42–60°C
Peters et al. 2000 [3]	Dog (x2)	830 nm (diode laser)	449–751 J 10–15 W 180–300 sec	1.5 T MRI control (thermometry)	4 hours and 24 hours	120–260 mm^3^	Feasibility of MRI guidance and thermal monitoring
Fuentes et al. 2009 [4]	Dog (x2)	980 nm (diode laser)	450 J 5 W 90 s	1.5 T MRI control (thermometry, cellular damage, HSP production and cell viability planification)	immediately after	>12 × 12 × 12 mm	Good correlation between cellular damage planification and histopathology
Stafford et al. 2010 [5]	Dog (x7) 5 without tumour 2 with orthotopic tumour	980 nm (diode laser)	462–3460 J 4–14.3 W 40–524 s	1.5 T MRI control (thermometry, cellular damage planification)	immediately after	12.4–26.7 × 11.4–15.5 mm (median: 19–13.7 mm)	Accuracy of MRI template guidance Excellent correlation of planification with histopathology
Colin et al. Marqa et al. 2011 [6, 7]	Rat (x10) with heterotopic tumour	980 nm (diode laser)	375 J 5 W 75 s	7.0 T MRI control (cellular damage planification)	48 hours	923–1125 mm^3^ (median: 974 mm^3^)	Reproducibility for one level of energy Good correlation of planification and histopathology
Fuentes et al. 2009 [4]	*Ex vivo* canine prostate in 1% agar gel	980 nm (diode laser)	240 J 8 W 30 s	1.5 T MRI control (thermometry, cellular damage planification)	—	—	MRI calibration for *in vivo* experiments
Lindner et al. 2010 [10]	Gelatine phantom With tumor target of 5 cm^3^	980 nm (diode laser)	—	1.5 T MRI control (thermometry) ultrasonography CT scan Fluoroptic temperature probes	—	—	MRI, US, CTS compatible phantom Good correlation between MRI and fluoroptic thermometry
Woodrum et al. 2010 [11]	Cadavers (x5) 3 fixed in Formaldehyde 2 fresh	980 nm (diode laser)	1800–3600 J 15–30 W 120 s	3.0 T MRI control (thermometry and cellular damage planification)	—	22–27 × 23–28 mm	Feasibility of transperineal 3.0 T MRI guidance and real-time control

**Table 2:** Clinical trials concerning focal laser ablation of prostate cancer.

Reference Year	Number of patients	Wavelength Type of laser source	Number of fibers	Energy (Joules) Power (Watt) Time (seconds)	Type of real-time imagery control	Adverse events	Visible dimension of thermal necrosis	Carcinologic results or conclusions
Amin et al. 1994 [12]	1 patient	805 nm (Diomed diode laser)	3	3000 J 2 W 500 sec	US and CT scan	Mild dysuria	Unknown	Feasibility of FLA Biopsies at 10 days: necrotic tissue in targeted area, cancer cells in other areas
Atri et al. 2009 [13] Lindner et al. 2009 [14]	12 patients	830 nm (Indigo diode laser)	1 or 2	2880 J 2–15 W (temperature control at 100°C) 720 sec	CEUS and fluoroptic temperature probes 7-day follow-up 1.5T MRI	Perineal discomfort (3 patients) Mild hematuria (2 patients) Hematospermia (2 patients) Fatigue (1 patient)	300–4000 mm^3^	Biopsies at 6 months: 67% of patients free of tumour in the targeted area 50% of patients free of disease
Raz et al. 2010 [15]	2 patients	980 nm (Visualase diode laser)	≥2	Unknown	3D 1.5 T MRI control (thermometry, cellular damage planification) and CEUS just after procedure 15-day follow-up 1.5T MRI	No adverse event	Unknown	Feasibility of immediately repeated therapy
Lindner et al. 2010 [8, 9]	4 patients	980 nm (Visualase diode laser)	2 or 3	3260–5900 J	CEUS and fluoroptic temperature probes 7-day 1.5T MRI control followed by radical prostatectomy	Not described	2500–4500 mm^3^	Strong correlation between MRI findings and vital stain histopathology images (Pearson's correlation index = 0.89)
Lindner et al. 2011 [16]	2 patients	980 nm (Visualase diode laser)	Unknown	Unknown	3D robotic 1.5 T MRI control (thermometry, cellular damage planification)	Improvement of IPSS score (1 patient) No change of IIEF-5 score	8700–9300 mm^3^	Safe and precise robotic guidance of laser fiber Possible oblique insertion angles to provide adequate dose
Woodrum et al. 2011 [17]	1 patient with local recurrence of prostate cancer after prostatectomy	980 nm (Visualase diode laser)	2	Unknown	3 T MRI control (thermometry, cellular damage planification)	No change of potency or continence	Unknown	Feasibility of FLA for local recurrence of prostate cancer
